# Supplementary material for: BiG-SCAPE 2.0 and BiG-SLiCE 2.0: scalable, accurate and interactive sequence clustering of metabolic gene clusters
Source: Nat Commun. 2026 Jan 24;17:2000. doi: 10.1038/s41467-026-68733-5 (PMC12936217; doi:10.1038/s41467-026-68733-5)
Supplement: Supplementary file 2 — Description of Additional Supplementary Files [file 41467_2026_68733_MOESM2_ESM.pdf]

Description of Additional Supplementary Files:

Supplementary Data 1: Major defining characteristics of the nine benchmarking datasets with curated GCF assignments. Characteristics include dataset names and assigned codes, the number of biosynthetic regions (# BGC), the number of curated families they were grouped into (# GCF), as well as how many of these families contain only one BGC (# Singleton GCFs) and if the dataset contains manually trimmed BGC regions (Trimmed). Additionally, a count and list of the unique antiSMASH BGC classes present in the dataset is shown (# Class; Classes)

Supplementary Data 2: Curated GCF assignments for benchmarking dataset B (Divergent). Seven GCFs contain BGCs that produce the same or highly similar compounds in relatively distant taxa. An additional 16 unrelated singleton GCFs were added as negative signals spanning across these taxa and compound classes.

Supplementary Data 3: Proportional and absolute runtimes of the major tasks (Input parsing, hmmscan, hmmalign, distance calculation and GCF calling) performed by BiG-SCAPE versions 1.1 and 2.0, at increasing input dataset sizes.

Supplementary Data 4: Random partitions of the antiSMASH database 4.0 used during performance benchmarks. Partitions cover a range of dataset sizes and are provided as a list of accessions and BGC filenames.
